# The Moderating Role of Ethical Leadership on Nurses' Green Behavior Intentions and Real Green Behavior

**DOI:** 10.1155/2021/6628016

**Published:** 2021-04-16

**Authors:** Miaomiao Li, Zhenxing Gong, Faheem Gul Gilal, Lyn M. Van Swol, Jifeng Xu, Fei Li

**Affiliations:** ^1^Beijing Information Science & Technology University, Beijing 100101, China; ^2^Liaocheng University, Liaocheng 252000, China; ^3^University of Wisconsin-Madison, Madison 53706, USA; ^4^Sukkur IBA University, Sukkur, Sindh 65200, Pakistan; ^5^Liaocheng Infectious Diseases Hospital, Liaocheng 252000, China

## Abstract

**Aim:**

This study is aimed at exploring the relationship between green behavior intentions and green behavior and analyzing the moderating role of ethical leadership in this relationship.

**Background:**

Nurses' green behavior can directly reduce costs and protect the natural environment and organizational sustainability by saving resources and energy. It is not clear how green behavior intention affects green behavior or how the positive influence of green behavior intention on green behavior can be enhanced. *Design and Methods*. This is a cross-sectional study, and the surveys are collected from 3 hospitals in China. Of the initial cohort of 489 nurses, 89.6% were female. There were 327 subjects (66.9%) aged 35 or less, 267 subjects (54.6%) with 10 years or less of work experience, and 220 unmarried subjects (44.9%). Data were collected from January to July 2018, using three surveys: green behavior intentions, green behavior, and ethical leadership.

**Results:**

Green behavior intentions impacted employee green behavior (*b* = 0.32, *t* = 5.37, *p* < 0.01). The interaction term for green behavior intentions and ethical leadership was significant (*b* = 0.28, *t* = 2.53, *p* ≤ 0.01); the conditional direct effect of green behavior intentions was only significant at a high level of ethical leadership (conditional effect = 0.53, SE = 0.16, *t* = 3.38, *p* < 0.01, 95% confidence interval of 0.22-0.84).

**Conclusion:**

The intention to engage in green behavior influences nurses' green behavior positively, and the relationship is stronger when ethical leadership is high in the organization than when ethical leadership is low. The results of this study can help both academics and practitioners to understand the micromechanism of environmentally sustainable development in more detail and to identify the mechanisms and boundary conditions of green behavioral intentions, green behavior, and ethical leadership.

## 1. Introduction

Environmentally sustainable development is a serious global problem facing all of humankind. Previous studies have shown that environmental pollution is largely caused by human activities [1]. The voluntary environmental protect action is considered to play the most important role in environmental pollution control and sustainable development; thus, organizational researchers and practitioners care about environmental issues [2], and the business idea of “being green” is now widely accepted. Prior research has found that the individual behavior is significantly related to organizational environmental performance [3], cost saving and competitive advantage, and waste reduction [4]. The effectiveness of hospital initiatives for sustainable development relies on nurses' behavior [5].

The energy consumption of hospitals in China is relatively high. The energy consumption of 500-bed general hospitals reaches 1400 TCE/year, and the energy consumption of hospitals accounts for 2.09% of the total hospital cost, and the energy consumption is on the rise. In addition, by the end of 2019, China had produced 843,000 tons of medical waste in 196 large- and medium-sized cities. In October 2019, the Ministry of Ecology and Environment of China issued specific guidance on the three outstanding problems in the current environmental management of hazardous waste, including weak environmental supervision capacity, unbalanced utilization and disposal capacity, and shortcomings in environmental risk prevention capacity. China has gradually formed a technical situation in which incineration and nonincineration disposal technologies coexist, but medical waste is still mainly disposed of, and the end control of medical waste is paid attention to while the reduction of medical waste from the source is ignored [6].

The hospital in China pays attention to environmental protection behaviors related to business activities but ignores the voluntary environmental protection behaviors of nurses, such as daily office environmental protection behaviors (turning off lights after use, not using disposable supplies, garbage classification, waste recycling, etc.), and green living behaviors (green travel, etc.) that have positive effects on environmental protection. The researchers invited future research on nurses' green behavior, which is defined as an element of environment-friendly behavior within organizational citizenship behavior and considered an essential component of organizational sustainable development that reflects the sense of responsibility of citizens at the ethical level [7]. Nurses' green behavior can directly reduce costs and protect the natural environment and organizational sustainability by saving resources and energy. Hence, an exploration of nurse and leader predictors of nurses' green behavior is needed [8].

Some previous research examined green behavior intention instead of actual green behavior [9]. It is not appropriate to equate green behavior intentions with green behavior and conclude that green behavior intention relates to green behavior positively, because intentions are often weakly related to enacted behavior, and the effect of interventions designed to change intentions on subsequent behavior is not as great as we may imagine. A meta-analysis showed that the relationship between propensity and behavior is quite small [10]. In addition, there have been few studies on the relationship between green behavior intentions and green behavior, and the findings of previous studies on the relationship between green behavior intentions and green behavior were not consistent. The effect of intention on recycling behavior was found to be very small (*β* = 0.07) in a hospital [11]; in contrast, Holland et al. found that the relationship between intention and recycling behavior was strong and positive in an office environment (*rs* between 0.48 and 0.64) [12]. These findings show that we cannot simply equate green behavior intention with green behavior and that the influence of green behavior intention on green behavior can differ among different samples. An important gap is that the majority of studies have focused on the positive or negative relationship between green behavior intentions and green behavior rather than the reason why their relationship is not consistent. The present study focused on how green behavior intention affects green behavior in Chinese hospitals and how the positive influence of green behavior intention on green behavior can be enhanced.

Nurses' green behavior reflects a sense of responsibility at an ethical level [13], and ethics is related to leadership directly in organizational life. Ethical leadership is a distinct leadership type. An ethical leader impacts the behavior of their subordinates by serving as a role model [14]. Ethical leaders are honest and impartial in their decisions [15]. Empirical studies have found that people who think more about morality and ethical issues tend to be more concerned about the well-being of others and engage in more proenvironment behaviors at work [16]. Since individuals work with high ethical leader, they will feel the approval of the leader for green behavior and demand the subordinates to be responsible for the green behavior [17]. If they translate their green behavior intention into real action, the leadership recognition they gain will be expanded. However, less has been examined about the role of ethical leadership in facilitating from intention to behavior. Some literature justifies the need for further research in this area [18]. We entail the investigation of this mechanism as an organization through ethical leaders can better implement green behavior practices, which can expand the promoting role of green behavior.

Ethical leaders influence nurses to engage in ethical behavior in the management process by setting an example (showing their behavior in line with ethical standards), taking morality as a guide, through a long-term stable behavior imitation and reinforcement process [15]. Though ethical leaders can influence employees' green behavior positively, how ethical leaders influence the relationship between nurses' green intention and behavior is an area that has not gained due attention of researchers [18]. With this background, this study has attempted to examine how ethical leaders moderate the relationship between nurses' green intention and behavior.

### 1.1. Green Behavior Intentions and Green Behavior

Green behavior includes behaviors such as turning off the lights when the nurse leaves, editing a file electronically rather than printing it out, using teleconferencing instead of traveling to a face-to-face meeting, or using waste paper to print a draft [7]. Although studies on the relationship between intention and behavior have not had consistent results, most prior research found a positive relationship between them, even if the correlation was not significant [12]. Individuals who set the goal to protect the environment and sustainable development should be more likely to show green behavior because setting a goal increases one's inner motivation. Ajzen argued that intention toward a behavior is a positive or negative feeling toward an objective object or the implementation of a specific behavior [19]. The more positive the intention toward an action, the stronger the behavior. The occurrence of green behavior among employees depends primarily on their intentions toward green behavior. The more positive the intention toward green behavior, the stronger the desire to adopt green behavior.

**Hypothesis 1 .**

Green behavior intentions are positively associated with employee green behavior.

### 1.2. The Moderating Role of Ethical Leadership

Ethical leadership is a model of leading and also guiding and molding nurses' ethical behavior. The leaders use various means to encourage their subordinates to engage in ethical behavior, such as subjective norms, perceived behavior control, and perceptual action control. Managers' actions speak louder than words [20]. “Subjective norm” refers to the pressure from influential individuals or groups in the process of making a decision to implement or not to implement a specific behavior, which reflects the influence of others on the individual's behavior decisions, mainly in the form of whether the subject thinks that a certain behavior should be performed. Research shows that managers' commitment to the environment is often superficial and formal [21]; therefore, it is vital to lead by action. Perceived behavior control refers to behavior that is not completely controlled by the will. It is the degree to which an individual perceives the difficulty or ease of performing a specific behavior and reflects the variables that may promote or reduce the implementation of the action based on his/her past experience and expectations [22]. The stronger the perceived behavior control, the easier to perform the behavior, and the stronger the intention of performing the behavior. Perceptual action control reflects the actual control situation of a behavior, and it can directly predict the possibility of actual behavior.

Ethical leaders are ethical people who are characterized by honesty, trustworthiness, and caring for subordinates. An ethical leader is also an ethical manager, who makes fair decisions based on ethical values, publicizes the importance of morality to subordinates, and standardizes subordinates' behaviors with rewards and punishments, so as to require subordinates to be responsible for the ethic of their own behaviors [23]. According to social learning theory, subordinates will regard leaders with power and status as role models to learn from. Individuals judge the ethic of their behaviors by summarizing the rewards/punishments received from their own behaviors or others' behaviors and adjust and regulate their own behaviors accordingly [24]. By using employees' example to learn from them, ethical leadership has realized its influence on the morality of subordinates' behavior.

Ethical leaders pay due attention to the principle of fairness when choosing how to treat their subordinates. Ethical leaders will pay special attention to procedural justice and interactive justice when allocating their energy resources [23]. For example, when a member is perceived to have green behavior intention, an ethical leader who pays attention to interactive justice will also make corresponding and equal efforts, so that the member can be motivated by the leader to a large extent [17]. In the case of a leader with a high level of ethical leadership, individuals are more likely to translate green behavioral intention into actions. Since individuals feel high ethical leadership, they will feel the approval of the leader for green behavior and demand the subordinates to be responsible for the behavior [17]. If they do not translate their green behavior tendency into action, the leadership recognition they gain will be reduced. Therefore, a high level of ethical leadership enhances the positive impact of green behavior orientation on green behavior. On the contrary, when the level of moral leadership is low, individuals find the gap between themselves and leadership requirements through upward comparison [23]. Combined with the perception of the unfairness of the leader, individuals will realize that it is difficult to gain the approval of the leader by relying on their own green behavior. Instead, they will feel more unfair and jealous. Therefore, a low level of moral leadership weakens the positive impact of green behavior orientation on green behavior.

For nurses, green behavior is an individual behavior consistent with environmentally sustainable development goals, and the positive green behavior of leaders encourages employee green behavior. Ethical leadership promotes nurses' green initiatives by sharing leaders' views on the environment with their subordinates, establishing organization value, and encouraging mutual awareness [25]. Ethical leaders show their values through their positive green behavior, and setting an example is a chance for managers to deliver value to subordinates [26]. When an organization formally implements a sustainable development plan, its importance is signaled by managers' active green behavior [26]. When managers engage in green behavior, nurses feel the support of managers for green behavior. The more active the green advocacy, the more employees perceive green behavior as being recognized, thus promoting nurses' green behavior. Ethical leadership can have a complementary effect to that of the green behavior intentions of nurses.

**Hypothesis 2 .**

Ethical leadership moderates the relationship between green behavior intentions and green behavior; that relationship is stronger when ethical leadership is high in the organization than when ethical leadership is low.

In brief, this study is aimed at exploring the relationship between green behavior intentions and green behavior and analyzing the moderating role of ethical leadership in this relationship.

## 2. Methods

### 2.1. Study Design and Procedure

This is a cross-sectional study, and the surveys are collected from 489 nurses in 3 hospitals in China. Of the initial cohort of 489 nurses, 89.6% (*n* = 438) were female, and 10.4% (*n* = 51) were male. As for their age, 66.9% (*n* = 327) were 26–30 years of age, and 33.1% (*n* = 162) were above 31 years old. Regarding their organizational tenure, 54.6% (*n* =268) had worked for less than 10 years and 55.4% (*n* = 271) for 11 years above. We received formal approval from the Ethics Committee for Research of the School of Business at Liaocheng University before conducting the survey.

We sought the consent of relevant leaders of the hospital and issued an anonymous questionnaire under the guidance of the director of the office. Then, the researcher described the aim of the research to the nurses in the meeting room. Nurses completed the survey anonymously. All the questionnaires were completed during nurses' work hours. The nurses were investigated by layer cluster sampling, stratified by general hospital units. According to the principle of convenience sampling, 489 valid questionnaires (completed in all items and excluding invalid answers, such as only one score provided for the entire questionnaire) were received.

This study adopted a single-factor test to the common method variance. When conducted principal component analysis with all factor fixed as one component, the factor with the most massive explanatory power was 31.22% that was not greater than 40%, confirming that there was no problem.

### 2.2. Measures

#### 2.2.1. Green Behavior Intentions

The green behavior intentions scale consisted of a three-item questionnaire developed by Norton et al., with items such as “I intend to perform pro-environmental behaviors while at work,” rated on a 5-point Likert scale. Researchers have validated the same scales in the Asian context [27]. As in previous studies, this study only considered the overall green behavior intention level and recorded the average value of all subjects as the whole level. Cronbach's alpha coefficient was 0.86.

#### 2.2.2. Ethical Leadership

The ethical leadership scale developed by Brown et al. [15] contains 10 statements, such as “My leader disciplines others in the unit who violate ethical standards,” which are rated on a 5-point Likert scale. Cronbach's interval coefficient was 0.87. Researchers have validated the same scales in the Asian context [17].

#### 2.2.3. Employee Green Behavior

The employee green behavior scale, developed by Norton et al. [7], contains five questions, such as “Thinking about your work today, to what extent did you avoid waste?” Cronbach's alpha coefficient was 0.96. Researchers have validated the same scales in the Asian context [28].

#### 2.2.4. Control Variables

Consistent with previous creativity research, we controlled for demographic variables such as age, gender, job tenure, and education.

### 2.3. Data Analysis and Availability

SPSS 22.0 was used for descriptive analysis, Pearson's correlation analysis, and regression analysis [29]. The SPSS PROCESS macro was used to calculate the moderation effect and conditional effects [30]. The data used to support the findings of this study are available from the corresponding author upon request.

## 3. Results

Based on preliminary data analysis, it was found that the fitting of each questionnaire index was good, the average variation extraction AVE value was between 0.66 and 0.70, and the combination reliability was between 0.91 and 0.95, both of which met the requirements of AVE > 0.5 and the combination reliability of CR > 0.5. On the whole, the convergence of each measurement scale is very good. Variance inflation factor (VIF) values of the variables are all in the range of 1.33 to 3.21, less than the critical value of 10, which means there is no common method bias problem. The results of discriminant validity test show that the three-factor model fits best, that is, the main research constructs all have good discriminant validity.

From the data in Table 1, it is evident that green behavior intentions were related to green behavior (*r* = 0.47, *p* < 0.01). Ethical leadership was also positively correlated with employee green behavior (*r* = 0.68, *p* < 0.01).

Table 2 shows that green behavior intentions associated with employee green behavior (*b* = 0.32, *t* = 5.37, *p* < 0.01), supporting H1. Ethical leadership is also associated with employee green behavior (*b* = 0.61, *t* = 2.56, *p* ≤ 0.01).

**Figure 1:**
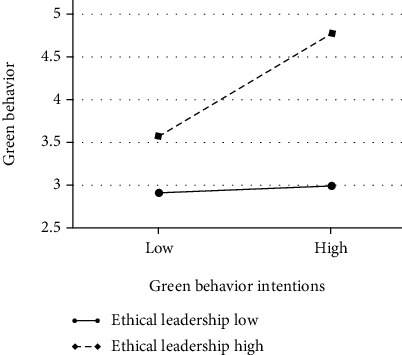
Simple slopes of green behavior intentions predicting employee green behavior at low (1 SD below M) and high (1 SD above M) levels of ethical behavior.

The interaction term for green behavior intentions and ethical leadership was significant (*b* = 0.28, *t* = 2.53, *p* ≤ 0.01).  Figure 1 illustrates the form of the interaction (Aiken & West, 1991).

Furthermore, to test the moderating role of ethical leadership, Preacher et al.'s statistical significance test was used. Specifically, we operationalized high and low levels of ethical leadership.  Table 3 shows that the conditional direct effect of green behavior intentions was stronger and significant at a high level of ethical leadership (conditional effect = 0.53, SE = 0.16, *t* = 3.38, *p* < 0.01, 95% confidence interval of 0.22-0.84) and at a moderate level of ethical leadership (conditional indirect effect = 0.32, SE = 0.12, *t* = 2.56, *p* < 0.01, 95% confidence interval of 0.07-0.56) but was positive and not significant at a low level of ethical leadership (conditional indirect effect = 0.10, SE = 0.12, *t* = 1.90, ns, 95% confidence interval of −0.12 to 0.33). Hence, H2 was supported.

As a precaution, we retested the hypothesis without including control variables [31]. The test model and the results were unchanged when the model was tested without age, gender, job tenure, or education included in the analysis.

## 4. Discussion

Climate change is largely caused by the adverse effects of human activities, and the success of environmental action often depends on the individual actions of employees [32]. The purpose of this study was to explore the effect of the intentions of green behavior on green behavior. This study was in the context of China, providing support for green-driven growth and entrepreneurship at the micro-level. As argued by Kim et al., to achieve sustainable development, the importance of green behavior must be realized at different levels (individuals, organizations, and countries). As green development has gained more and more acceptance worldwide, its application at the microlevel in the Chinese context can lead to new progress for sustainable development.

### 4.1. Theoretical Implications

First, our study explains if green behavior intentions lead to green behavior in China. This study extends the research in the field of green behavior. Our research focused on the influencing mechanism of green development at the microlevel, helping to fill the gap in the existing research, which focused primarily on the macro and middle levels. This study confirmed the impact of green behavior intention on green behavior. We found that green behavior intentions promote nurses' green behavior. This finding is consistent with the theory of planned behavior [19]. Individuals' behavior is determined by their intentions, which are directly impacted by their attitudes. Meanwhile, individuals' attitudes are also influenced by their expectations and evaluations of behavioral results (result beliefs); that is, individuals' attitudes are influenced by their beliefs about the results of behaviors and indirectly influence their behavior through behavioral intentions. This result is also consistent with Holland et al., who found a positive relationship between intentions and recycling behavior [12].

Second, we further clarify why high green behavior intentions not always lead to green behavior and analysis the relationships among green behavior intentions, ethical leadership, and green behavior. We found that green behavior intentions and ethical leadership complement each other and that green behavior intentions are more important for employees who work under a lower level of ethical leadership. Moreover, there is more employee green behavior among employees who work under a higher level of ethical leadership than employees who work under a lower level of ethical leadership. We found that the impact coefficient of green behavior intentions on green behavior was stronger for employees who worked under a higher level of ethical leadership. Ethical leadership plays a moderating role in the relationship between green behavior intentions and green behavior, and green behavior intentions and ethical leadership complement each other. Employee participation is a key factor in promoting environmental sustainability [32]. Organizational managers influence many behavior outcomes, such as organizational safety and environmental performance [33]. Ethical leaders set an example through their green behavior and influence the sustainable development of organizations through their environmental ethical commitments. Environmental ethics leaders tend to have a collectivist spirit that transcends their own interests. They usually extend their commitment to environmental ethics, to the organization's environmental management practices, and even to the sustainable development of the environment to influence employees' green behaviors.

### 4.2. Practical Implications

The results could provide a new perspective for environmental pollution management and help leaders achieve their green-driven growth and development. This research can provide a new perspective—focused on management practice—for sustainable development research. The intermingled ethical and social responsibility of enterprise managers is an important factor that restricts further development of enterprise environmental performance. Hospitals should list environmental ethics culture as an important content of corporate culture, so that the publicity of environmental ethics is everywhere and the awareness of environmental ethics is deeply rooted in the hearts of the people. Hospitals need to be fully permeated with the concept of environmental protection. Hospitals can use bulletin boards, work briefings, environmental knowledge manuals, environmental microfilms, environmental moral public welfare volunteer activities, etc., to convey national and enterprise laws, policies, systems, and regulations related to environmental protection [34]. Leaders need to advocate the staff to establish the concept of environmental conservation, environmental protection, and sustainable development, cultivate the environmental protection ethics of the enterprise and its members, and establish a sense of social responsibility and mission.

The ethical level and moral behavior of managers guide the overall ethical level of enterprises, which influences the sustainable development of organizations. To improve employees' green behavior, managers should establish the ethical concept of environmental protection and seek to lead incorruptibly, giving play to the role of environmental ethical models and incorporating strict environmental ethics into their personal behavior to build a strong corporate environmental moral atmosphere, improving the level of environmental protection in enterprise management. Managers should avoid blindly pursuing short-term economic interests and should have a long-term vision and a high degree of social responsibility. Leaders need to take environmental ethical standards as an important aspect of their organization's social responsibility, identify them as development directions and goals of the enterprise, integrate them into the core values of the enterprise, and actively advocate for an environmental ethical culture. They should set up the environmental protection, moral standards of energy conservation and emission reduction, effective utilization of resources, environmental protection, and enhancement of environmental protection awareness. These measures can improve the level of environmental protection ethics of organizations and help achieve the environmental protection goals of enterprises.

### 4.3. Limitations and Future Research Directions

There are a few limitations to our study. First, all of the data were from self-assessment questionnaires. Participants self-reported their green behavior, which is more subjective than regulators' or peers' evaluations, comments, or records. Self-reports can be influenced by social desirability. Hence, in addition to self-reporting, future research could attempt to collect objective data. Second, we used general items to measure employees' green behavior intentions, but specific items to measure actual green behavior. The relationship between these variables is likely to be strengthened if specific items are used for both measures. Recent studies have used more specific concepts of green behavior than our study. For example, one study distinguished between demanded (i.e., formal requirements) and proactive forms of green behavior and showed that they were associated with different antecedent variables [35]. Future research on green behavior could also distinguish between active behavior and behavior that is not done.

## 5. Conclusions

In conclusion, this research demonstrates the importance of further considering the moderation role of ethical leadership in the relationship between green behavior intentions and green behavior. The intention to engage in green behavior associates with nurses' green behavior positively, and this relationship is stronger when ethical leadership is high in the hospital than when ethical leadership is low. The results of this study can help both academics and practitioners to understand the micromechanism of environmentally sustainable development in more detail and to identify the mechanisms and boundary conditions of green behavioral intentions and green behavior.

## Figures and Tables

**Table 1:** Means, standard deviations, and correlation of all measures.

Variable	Mean	SD	1	2	3
1. Green behavior intentions	2.61	0.65	—		
2. Ethical leadership	3.03	0.74	0.61^∗∗^	—	
3. Employee green behavior	3.37	0.96	0.47^∗∗^	0.68^∗∗^	—

^1^Note. *n* = 489; ^∗^*p* < 0.05, ^∗∗^*p* < 0.01.

**Table 2:** Hierarchical regression results about direct and moderation effect.

Variable	*β*	SE	*t*	*p*	LLCI	ULCI
Green behavior intentions	0.61	0.11	5.37	<0.01	0.38	0.83
Ethical leadership	0.32	0.12	2.56	0.01	0.07	0.56
Interaction	0.28	0.08	3.53	<0.01	0.12	0.43

^1^Note. *n* = 489; ^∗^*p* < 0.05, ^∗∗^*p* < 0.01.

**Table 3:** Exploratory analyses of the moderating effects of ethical leadership on the relationship between green behavior intentions and employee green behavior.

Variable	Moderator level	Mean	Effect	SE	t	p	LL 95% CI	UL 95% CI
Ethical leadership	Low (M–1SD)	-0.77	0.10	0.12	0.90	0.37	-0.12	0.33
	Moderate level	0	0.32	0.12	2.56	0.01	0.07	0.56
	High (M+1SD)	0.77	0.53	0.16	3.38	0.01	0.22	0.84

## Data Availability

The data used to support the findings of this study are available from the corresponding author upon request.

## Additional Points

*Implications for Nursing Management*. Leaders should promote nurses' green initiatives by sharing leaders' views on the environment with their subordinates, establishing organization value, and encouraging mutual awareness.
